# Artificial intelligence-based diagnostic model for identifying PTPRZ1-MET fusion in a clinically defined secondary glioblastoma cohort

**DOI:** 10.1186/s43556-026-00529-x

**Published:** 2026-08-04

**Authors:** Jin-Hao Zhang, Ji Shi, Han-Xiao Zhou, Wen-Lu Tan, Ying Zhang, Tao Jiang, Zheng Zhao

**Affiliations:** 1 Department of AI and Biomedical Information, Beijing Neurosurgical Institute, Capital Medical University, Beijing, 100070 China; 2 Department of Neurosurgery, Beijing Tiantan Hospital, Capital Medical University, Beijing, 100070 China; 3 Department of Neurosurgery, Liaoning Cancer Hospital & Institute, Cancer Hospital of Dalian University of Technology, Cancer Hospital of China Medical University, Shenyang, 110042 China; 4 Genome Atlas (CGGA/AGGA) Network, Beijing, 100070 China; 5 Chinese Neuro-Oncology Genome Atlas (CGGA-CNS) Network, Beijing, 100070 China

**Keywords:** Glioma, PTPRZ1-MET fusion, Biomarker, Machine learning, Diagnosis

## Abstract

Glioblastoma (GBM) is an aggressive and highly lethal brain tumor. Secondary glioblastoma (sGBM), which arises through malignant progression from lower-grade diffuse glioma, represents a clinically and biologically distinct subset of GBM. In this disease context, the protein tyrosine phosphatase receptor type Z1–mesenchymal-epithelial transition factor (PTPRZ1-MET; ZM) fusion has emerged as a recurrent oncogenic driver associated with adverse clinical outcomes. In this study, we analyzed 159 patients with sGBM, including 15 ZM-positive and 144 ZM-negative cases, and confirmed that ZM-positive tumors were associated with significantly shorter overall and progression-free survival. Transcriptomic profiling identified 359 genes upregulated in ZM-positive tumors, with enrichment in cell-cycle regulation and mitotic spindle-related pathways. To explore surrogate biomarkers associated with ZM fusion status, we benchmarked eight machine-learning classifiers and retained XGBoost as the primary feature-prioritization model. MET, PCDHGA3, and FAM3C emerged as the most informative biomarkers, and the fixed three-gene panel showed stable discriminative performance across cross-validation, nested evaluation, feature-pool sensitivity analyses, repeated random seeds, and class-weighted modeling. Protein-level validation in an independent formalin-fixed, paraffin-embedded (FFPE) cohort using multiplex and conventional chromogenic immunohistochemistry supported the pathology-compatible detection of elevated MET, PCDHGA3, and FAM3C expression in ZM-positive tumors. Collectively, these findings support a robust three-gene molecular signature that may facilitate the identification and stratification of ZM fusion-positive sGBM and provide biological insights with potential translational relevance for precision glioma diagnosis.

**Supplementary Information:**

The online version contains supplementary material available at 10.1186/s43556-026-00529-x.

## Introduction

Glioblastoma is an aggressive primary malignant tumor of the central nervous system, characterized by diffuse infiltration, rapid clinical deterioration, and intrinsic resistance to conventional therapies [1]. Despite maximal safe resection followed by radiotherapy and temozolomide chemotherapy, the prognosis remains poor, with median overall survival limited to 14–18 months and a 5-year relative survival rate of approximately 6.8% [2–4]. The 2021 World Health Organization (WHO) Classification of Central Nervous System Tumors established a biologically driven taxonomy for diffuse gliomas, emphasizing integrated molecular diagnosis based on key alterations such as IDH mutation status [5, 6]. Although the term secondary glioblastoma (sGBM) has been refined in contemporary molecular classification, it remains a clinically relevant historical concept describing glioblastoma that develops through malignant progression from lower-grade diffuse glioma. Molecular alterations associated with this progression, including IDH1/2 and TP53 mutations, ATRX loss, CDKN2A/B homozygous deletion, and oncogenic gene fusions, contribute to high-grade transformation and patient prognosis [7].

Beyond these canonical drivers, gene fusions have emerged as a critical class of oncogenic events across solid tumors, often serving as both molecular diagnostic markers and therapeutic targets [8]. Well-established examples, such as EML4-ALK and ROS1 fusions in lung adenocarcinoma or TMPRSS2-ERG fusion in prostate cancer, illustrate the profound clinical impact of fusion-driven oncogenesis [9–11]. In gliomas, recurrent FGFR3-TACC3 fusions induce aberrant kinase activation and mitotic spindle dysfunction, underscoring the biological significance of fusion events in gliomagenesis [12]. Our previous genomic analyses identified the protein tyrosine phosphatase receptor type Z1–mesenchymal-epithelial transition factor (PTPRZ1-MET; ZM) fusion as a recurrent oncogenic alteration enriched in tumors historically referred to as secondary glioblastomas [13, 14]. The ZM fusion aberrantly activates MET signaling, accelerates malignant progression, and is associated with inferior clinical outcomes. Importantly, these mechanistic insights have yielded translational advances: the selective MET inhibitor Vebreltinib (PLB-1001), developed based on ZM fusion biology, has demonstrated antitumor activity in preclinical glioma models and Phase I-III clinical evaluations in patients with ZM-positive sGBM [14–16]. Together, these findings establish ZM fusion as a biologically and clinically relevant molecular event with potential therapeutic implications.

Despite these advances, the identification of ZM-positive tumors in clinical practice remains challenging. Current detection strategies rely predominantly on sequencing-based or RT-PCR/Sanger-based methods, which are definitive but require high-quality nucleic acids, specialized platforms, and additional turnaround time [17]. These requirements may limit their implementation in routine neuropathological workflows. Moreover, no validated surrogate biomarker panel is currently available to support the screening or prioritization of ZM-positive tumors in settings where advanced genomic testing is not readily accessible.

To address these limitations, we performed an integrative analysis combining clinical outcomes, transcriptomic profiling, and machine-learning–based biomarker prioritization in a cohort of 159 patients with sGBM. We assessed the prognostic significance of ZM fusion, delineated ZM-associated transcriptional programs, and benchmarked eight machine-learning classifiers to identify transcriptomic biomarkers capable of distinguishing ZM-positive from ZM-negative tumors, with XGBoost used as the primary feature-prioritization model. Candidate biomarkers were further validated at the protein level using multiplex and conventional chromogenic immunohistochemistry in an independent cohort. Together, these analyses provide a comprehensive characterization of ZM fusion biology in sGBM and establish a practical molecular signature with potential diagnostic and translational utility.

## Results

### Clinical characteristics and ZM fusion variant landscape in sGBM

**Fig. 1:**
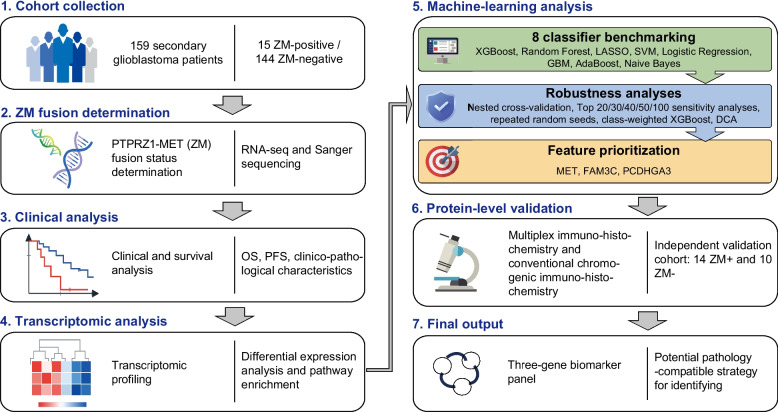
Study workflow. The overall study design included patient cohort collection, ZM fusion determination, clinical and survival analyses, transcriptomic profiling, machine-learning-based biomarker prioritization, robustness analyses, and protein-level validation in an independent formalin-fixed, paraffin-embedded (FFPE) cohort

The overall study workflow included patient cohort collection, ZM fusion determination, clinical and survival analyses, transcriptomic profiling, machine-learning-based biomarker prioritization, robustness analyses, and protein-level validation in an independent cohort (Fig. 1). A total of 159 patients with sGBM were included in this retrospective cohort, comprising 15 ZM-positive and 144 ZM-negative cases. Baseline demographic and clinical characteristics are summarized in Table 1. The mean age at diagnosis was comparable between the two groups (37.33 ± 6.91 years for ZM-positive vs. 41.86 ± 9.59 years for ZM-negative). Sex distribution, extent of resection, and tumor laterality also showed no significant differences. Although tumors involved multiple anatomical regions, ZM-positive cases demonstrated a relatively higher proportion of frontal lobe (86.7%) and temporal lobe (53.3%) involvement compared with ZM-negative cases (61.8% and 36.8%, respectively).

**Table 1:** Patient clinical characteristics

	**Total** **(*n* = 159)**	**ZM-positive** **(*n* = 15)**	**ZM-negative** **(*n* = 144)**
Age at diagnosis			
Mean	41.43 ± 9.47	37.33 ± 6.91	41.86 ± 9.59
Range	8—65	29—51	8—65
Male sex – no. (%)	102 (64.2%)	10 (66.7%)	92 (63.9%)
Extent of resection – no. (%)			
Subtotal resection	35 (22.0%)	5 (33.3%)	30 (20.8%)
Total resection	120 (75.5%)	9 (60.0%)	111 (77.1%)
Unknown/missing	4 (2.5%)	1 (6.7%)	3 (2.1%)
Tumor location – no. (%)			
Frontal lobe	102 (64.2%)	13 (86.7%)	89 (61.8%)
Parietal lobe	22 (13.8%)	0	22 (15.3%)
Temporal lobe	61 (38.4%)	8 (53.3%)	53 (36.8%)
Other	4 (2.5%)	0	4 (2.8%)
Laterality – no. (%)			
Left	69 (43.4%)	6 (40.0%)	63 (43.8%)
Midline	6 (3.8%)	1 (6.7%)	5 (3.5%)
Right	70 (44.0%)	7 (46.7%)	63 (43.8%)
Unknown/missing	14 (8.8%)	1 (6.7%)	13 (9.0%)

Values are presented as mean ± standard deviation or number (%). Extent-of-resection and laterality categories are mutually exclusive. Extent-of-resection information was unavailable for 4 patients, and laterality information was unavailable for 14 patients. Tumor-location categories are not mutually exclusive because some tumors involved more than one anatomical region

**Fig. 2:**
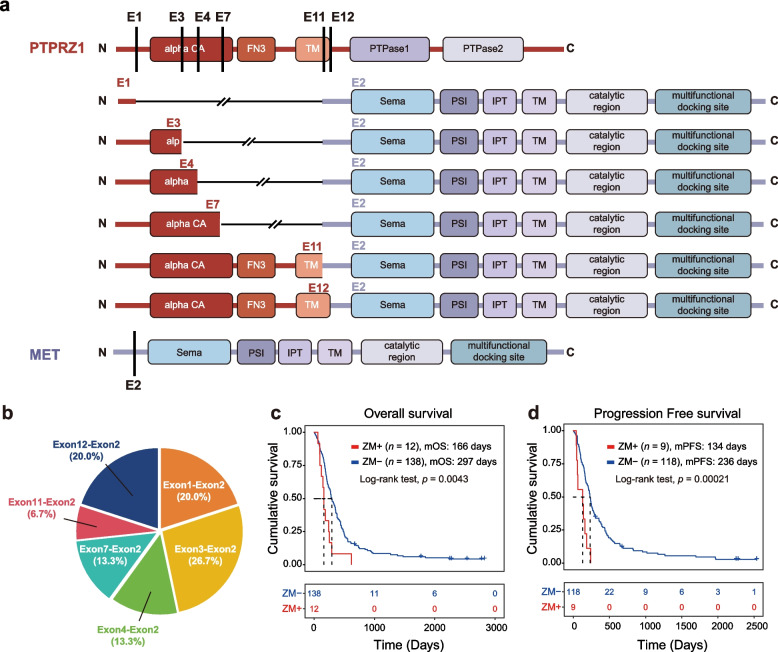
Landscape of PTPRZ1-MET (ZM) fusion variants and clinical outcomes in secondary glioblastoma (sGBM). **a** Schematic representation of the six identified PTPRZ1-MET fusion variants. PTPRZ1-derived regions are shown in red and MET-derived regions are shown in blue/purple. The schematic shows PTPRZ1 breakpoints at exons 1, 3, 4, 7, 11, and 12, all fused to MET exon 2. Protein domains are labeled, including alpha carbonic anhydrase-like domain (alpha CA), fibronectin type III domain (FN3), transmembrane domain (TM), PTPase domains, Sema domain, PSI domain, IPT domain, catalytic region, and multifunctional docking site. **b** Distribution of the six ZM fusion subtypes in the cohort. Different colors indicate different fusion subtypes, and percentages indicate the proportion of each subtype among ZM-positive tumors, including exon 3–exon 2 (26.7%), exon 1–exon 2 (20.0%), exon 12–exon 2 (20.0%), exon 7–exon 2 (13.3%), exon 4–exon 2 (13.3%), and exon 11–exon 2 (6.7%). **c** Kaplan–Meier overall survival curves comparing ZM-positive and ZM-negative patients. Red and blue curves represent ZM-positive and ZM-negative patients, respectively. The number-at-risk table below the plot shows the number of patients remaining at risk at each time point. Statistical significance was assessed using the log-rank test (*p* = 0.0043). **d** Kaplan–Meier progression-free survival curves comparing ZM-positive and ZM-negative patients. Red and blue curves represent ZM-positive and ZM-negative patients, respectively. The number-at-risk table below the plot shows the number of patients remaining at risk at each time point. Statistical significance was assessed using the log-rank test (*p* = 0.00021)

RNA-seq–based fusion detection identified six distinct PTPRZ1-MET fusion variants among the 15 ZM-positive tumors, with representative fusion events further validated by RT-PCR followed by Sanger sequencing (Fig. 2a). All variants shared a common breakpoint at MET exon 2, whereas PTPRZ1 breakpoints were distributed across exons 1, 3, 4, 7, 11, and 12. Four fusion types–PTPRZ1 exon 4–MET exon 2, exon 7-exon 2, exon 11-exon 2, and exon 12-exon 2–were newly discovered in this cohort. Among all detected variants, the most frequent were exon 3-exon 2 (26.7%), exon 1-exon 2 (20.0%), and exon 12-exon 2 (20.0%) fusions (Fig. 2b).

### ZM fusion is associated with poor clinical outcomes

To evaluate the prognostic impact of the ZM fusion, survival outcomes were compared between ZM-positive and ZM-negative patients. For OS analysis, 150 patients had evaluable survival information, including 143 deaths and 7 censored observations. For PFS analysis, 127 patients had evaluable progression information, including 122 progression/death events and 5 censored observations. Kaplan–Meier analysis demonstrated that the presence of the ZM fusion was associated with markedly worse prognosis. The median overall survival (OS) was 166 days for ZM-positive patients compared with 297 days for ZM-negative patients (*p* = 0.0043, log-rank test; Fig. 2c). A similar trend was observed for progression-free survival (PFS), with median values of 134 days and 236 days in the ZM-positive and ZM-negative groups, respectively (*p* = 0.00021, log-rank test; Fig. 2d). These findings indicate that the ZM fusion serves as a strongly adverse prognostic factor in sGBM.

### Association of ZM fusion with clinical characteristics

**Fig. 3:**
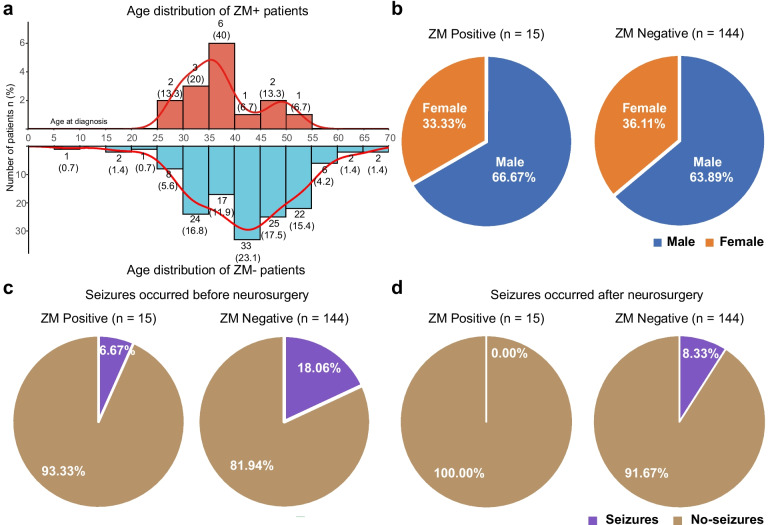
Clinical characteristics associated with ZM fusion status in sGBM. **a** Age distribution of ZM-positive and ZM-negative patients. ZM-positive patients were mainly concentrated in younger to middle-adult age groups, whereas ZM-negative patients showed a broader age range. **b** Sex distribution of ZM-positive and ZM-negative patients. **c** Preoperative seizure occurrence in ZM-positive and ZM-negative groups. Seizures were less frequently observed in ZM-positive patients before neurosurgery. **d** Postoperative seizure occurrence in ZM-positive and ZM-negative groups. No postoperative seizures were observed in the ZM-positive cohort, whereas a small proportion of ZM-negative patients experienced postoperative seizures

To further delineate the clinical distribution of ZM fusion beyond the baseline features summarized in Table 1, we examined its association with age, sex, and seizure presentation. The age distribution showed that ZM-positive patients were mainly concentrated in younger to middle-adult age groups, whereas ZM-negative patients showed a broader age range (Fig. 3a). The sex distribution was similar between ZM-positive and ZM-negative patients, with males accounting for 66.67% and 63.89% of cases, respectively (Fig. 3b).

Because seizures are a common presenting symptom in diffuse gliomas, we next assessed seizure occurrence in relation to ZM fusion status. Preoperatively, seizures were reported less frequently in ZM-positive patients (6.67%) than in ZM-negative patients (18.06%) (Fig. 3c). Moreover, no postoperative seizures were observed in the ZM-positive group, whereas a small proportion of ZM-negative patients continued to experience seizures (Fig. 3d).

Taken together, these observations suggest that ZM-positive tumors may represent a clinically distinct subgroup that preferentially occurs in younger patients and presents with a lower seizure burden, potentially reflecting differences in cortical involvement or tumor growth dynamics.

### ZM fusion-positive tumors display a proliferation-dominant transcriptomic program

**Fig. 4:**
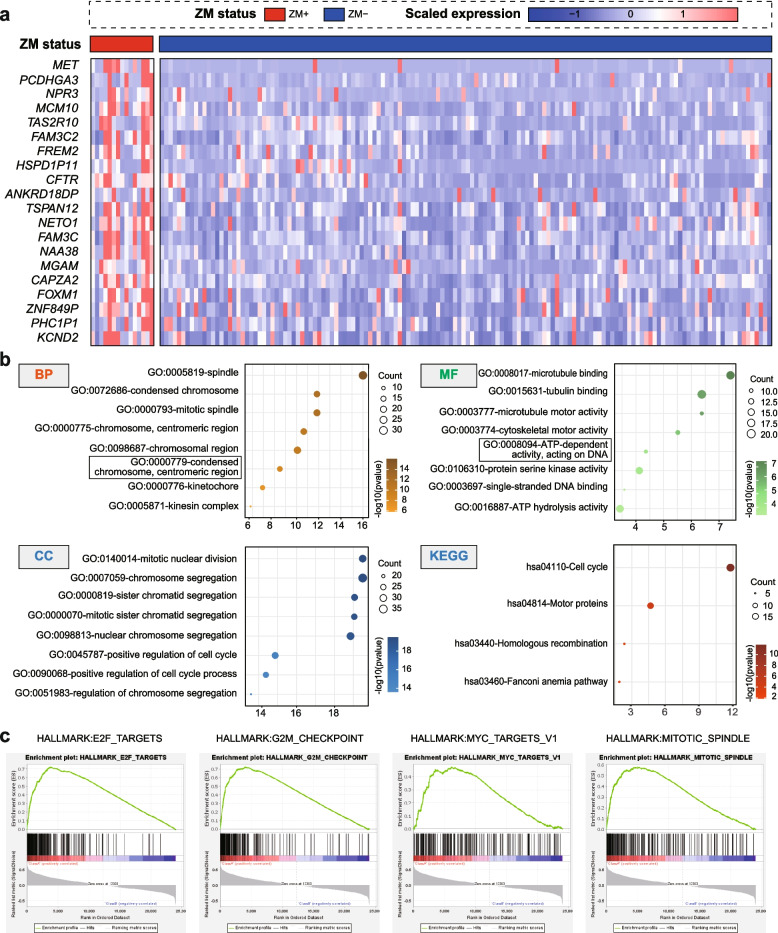
Transcriptomic profiling identifies a distinct molecular signature in ZM-positive tumors. **a** Heatmap of the top 20 upregulated genes in ZM-positive tumors. Gene expression was z-score normalized across samples. The top annotation bar indicates ZM fusion status, with red representing ZM-positive tumors and blue representing ZM-negative tumors. In the heatmap, red and blue indicate higher and lower relative expression, respectively. **b** Gene Ontology (GO) and Kyoto Encyclopedia of Genes and Genomes (KEGG) enrichment summary of the 359 upregulated genes in ZM-positive tumors. Enrichment results are shown for biological process (BP), molecular function (MF), cellular component (CC), and KEGG pathways. Dot size indicates the number of enriched genes, and dot color indicates − log10(P value). **c** Gene Set Enrichment Analysis (GSEA) showing representative hallmark gene sets enriched in ZM-positive tumors, including E2F targets, G2M checkpoint, MYC targets V1, and mitotic spindle. Green curves indicate running enrichment scores, and black vertical lines indicate the positions of genes from each gene set within the ranked gene list

Given the marked survival differences between ZM-positive and ZM-negative patients, we next investigated the transcriptomic features that may underlie these prognostic disparities. Gene-wise differential expression screening identified 359 upregulated candidate genes in ZM-positive tumors with fold change > 2 and nominal *p* < 0.05. A heatmap of the top 20 upregulated genes showed clear separation between ZM-positive and ZM-negative samples (Fig. 4a). To determine whether this broader ZM-associated transcriptional program was influenced by potential age or sex imbalance, we further evaluated the 359 candidate genes using unadjusted and age/sex-adjusted *limma* models. After covariate adjustment, all 359 genes retained a positive ZM-associated effect and 358 remained nominally significant in the age/sex-adjusted model, supporting the robustness of the ZM-upregulated transcriptional program (Table S1).

Functional enrichment analyses revealed that these upregulated genes were strongly associated with proliferative and cell cycle-related processes. Enriched Gene Ontology (GO) terms included chromosome segregation, mitotic spindle organization, kinetochore assembly, and microtubule motor activity, while Kyoto Encyclopedia of Genes and Genomes (KEGG) pathways prominently featured cell cycle regulation and homologous recombination (Fig. 4b). Consistent with these findings, Gene Set Enrichment Analysis (GSEA) showed significant enrichment of hallmark gene sets including *E2F *targets, *G2M *checkpoint, *MYC *targets, and mitotic spindle signaling in ZM-positive tumors (Fig. 4c).

Collectively, these results delineate a proliferation-dominant transcriptomic program in ZM-positive sGBM, providing a biological basis for their more aggressive clinical behavior.

### Machine-learning analysis identifies a three-gene signature associated with ZM fusion status

**Fig. 5:**
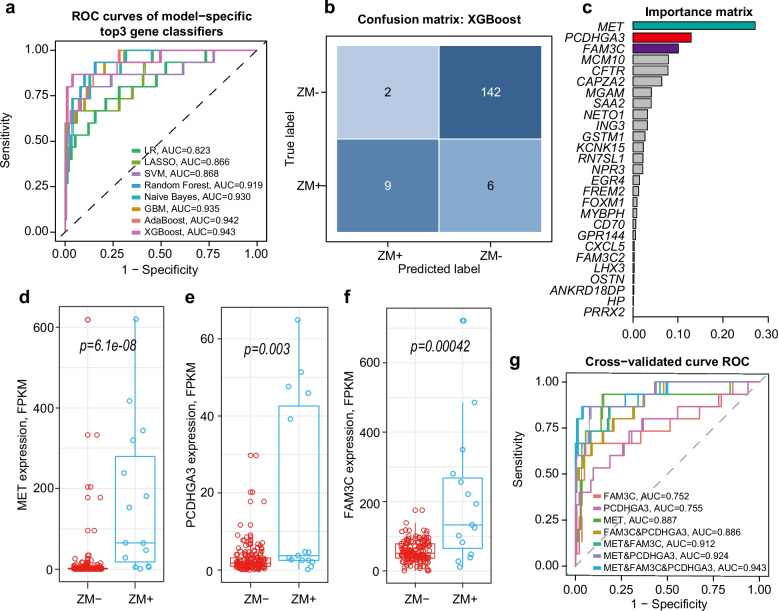
Machine-learning analysis identifies a three-gene transcriptomic signature associated with ZM fusion status. **a** Cross-validated ROC curves of eight model-specific three-gene classifiers generated from the Top40 candidate gene set. The AUC values of each classifier are shown in the plot. **b** Confusion matrix of the representative XGBoost model at the prespecified probability threshold of 0.5. **c** Gain-based feature-importance ranking from the XGBoost model, identifying MET, PCDHGA3, and FAM3C as the top-ranked candidate biomarkers. **d–f** Expression levels of MET, PCDHGA3, and FAM3C in ZM-positive and ZM-negative tumors. Each dot represents an individual patient sample, and statistical significance was assessed using the Mann–Whitney U test. **g** Cross-validated ROC curves evaluating single-gene, pairwise, and combined three-gene XGBoost classifiers. The combined MET/FAM3C/PCDHGA3 panel showed the highest discriminative performance among the tested combinations

To evaluate the diagnostic potential of ZM-associated transcriptomic biomarkers, we first benchmarked eight machine-learning classifiers using the Top40 candidate gene set. For each classifier, model-specific top-ranked features were selected from the Top40 candidate gene set to construct the corresponding three-gene classifier. In representative cross-validated ROC analyses, XGBoost achieved one of the highest discriminative performances among the evaluated algorithms, with an AUC of 0.943, followed closely by AdaBoost (AUC = 0.942), GBM (AUC = 0.935), naive Bayes (AUC = 0.930), random forest (AUC = 0.919), SVM (AUC = 0.868), LASSO (AUC = 0.866), and logistic regression (AUC = 0.823) (Fig. 5a and Table S2). The XGBoost-based confusion matrix showed high specificity and moderate sensitivity in the representative cross-validation run, correctly identifying 142 of 144 ZM-negative tumors and 9 of 15 ZM-positive tumors (Fig. 5b and Fig. S1a). Given its competitive performance and interpretable gain-based feature-importance framework, XGBoost was retained as the primary feature-prioritization model. To further assess the potential clinical utility of the XGBoost model, we performed decision curve analysis using the cross-validated predicted probabilities. The XGBoost model showed a higher net benefit than both the “all” and “none” strategies within the evaluated threshold range, supporting its potential value as an auxiliary strategy for prioritizing tumors with a higher likelihood of ZM fusion for further confirmation (Fig. S1b).

Gain-based feature-importance analysis of the XGBoost model prioritized MET, PCDHGA3, and FAM3C as the most informative candidate biomarkers (Fig. 5c). Consistent with the feature-prioritization results, all three genes showed significantly higher expression in ZM-positive tumors than in ZM-negative tumors (Fig. 5d-f). Consistent with the program-level robustness analysis, we further examined whether the three XGBoost-prioritized biomarkers remained upregulated after adjustment for age and sex. MET, PCDHGA3, and FAM3C all retained significantly higher expression in ZM-positive tumors in the age/sex-adjusted *limma* models, with directions of effect consistent with the unadjusted analysis (Table S3). These results indicate that the elevated expression of the three prioritized biomarkers was not solely attributable to age or sex imbalance between the two groups.

To assess whether the combined panel provided additional discriminative value beyond individual markers, we further constructed XGBoost classifiers using each single gene, each pairwise gene combination, and the combined three-gene panel. Among single-gene models, MET showed the strongest individual performance (AUC = 0.887), whereas FAM3C and PCDHGA3 showed moderate discriminative ability (AUC = 0.752 and 0.755, respectively). Pairwise combinations improved model performance, particularly MET/PCDHGA3 (AUC = 0.924) and MET/FAM3C (AUC = 0.912). The combined MET/FAM3C/PCDHGA3 panel achieved the highest AUC among the tested XGBoost combinations (AUC = 0.943), supporting the incremental value of integrating all three biomarkers (Fig. 5g).

To further reduce potential feature-selection leakage, we performed fully nested cross-validation, in which differential expression filtering, Top40 candidate gene selection, feature prioritization, hyperparameter tuning, and model training were conducted separately within each outer training fold. In this conservative analysis, the training-fold Top40 model yielded an outer-loop AUC of 0.860, with a sensitivity of 0.400, specificity of 1.000, PPV of 1.000, and NPV of 0.941 at the prespecified 0.5 threshold. The training-fold-selected Top3 model achieved an AUC of 0.890, with a sensitivity of 0.267, specificity of 0.986, PPV of 0.667, and NPV of 0.928. The fixed MET/PCDHGA3/FAM3C panel showed the highest AUC among the nested analyses (AUC = 0.938), with a sensitivity of 0.467, specificity of 1.000, PPV of 1.000, and NPV of 0.947. These results indicate that the three-gene panel is best interpreted as a high-specificity biomarker signature for prioritizing tumors with a high probability of ZM fusion, rather than as a high-sensitivity screening assay (Fig. S1c and Table S4).

We next assessed the robustness of this biomarker selection strategy. Sensitivity analyses using alternative candidate gene-set sizes, including Top20, Top30, Top40, Top50, and Top100, showed that the overall XGBoost performance remained stable across feature-pool sizes and random seeds (Fig. S1d). MET was consistently retained as a top-ranked feature, whereas PCDHGA3 and FAM3C were also repeatedly recovered among high-priority features in Top30 and larger candidate pools (Table S5). In addition, to account for the unequal number of ZM-positive and ZM-negative cases, we evaluated class-weighted XGBoost using the fixed MET/PCDHGA3/FAM3C panel across 50 repeated runs. The class-weighted model assigned a weight of 9.6 to ZM-positive samples and 1 to ZM-negative samples. Compared with the unweighted model, class-weighted XGBoost achieved a slightly higher mean ROC-AUC (0.927 ± 0.018 vs. 0.923 ± 0.015), improved sensitivity (0.669 ± 0.077 vs. 0.564 ± 0.084), and improved balanced accuracy (0.815 ± 0.037 vs. 0.774 ± 0.041). This increase in sensitivity was accompanied by a modest reduction in specificity (0.961 ± 0.008 vs. 0.984 ± 0.010) and PR-AUC (0.762 ± 0.031 vs. 0.777 ± 0.034). These results indicate that class weighting improved the detection of ZM-positive tumors while preserving comparable overall discriminative performance, supporting the robustness of the three-gene panel under imbalance-aware modeling (Table S6).

Collectively, these findings indicate that the combined expression profile of MET, PCDHGA3, and FAM3C defines a stable composite molecular signature for prioritizing tumors with a higher likelihood of ZM fusion in sGBM.

### Protein-level validation supports biomarker overexpression in ZM-positive tumors

**Fig. 6:**
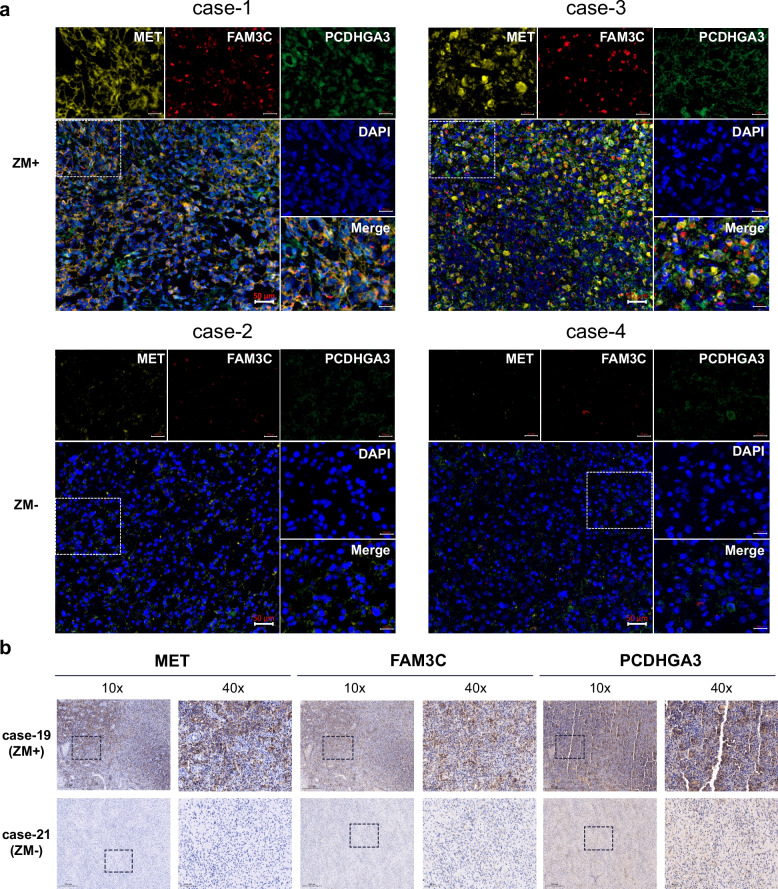
Protein-level validation of MET, PCDHGA3, and FAM3C expression in ZM-positive and ZM-negative tumors.** a** Representative multiplex immunofluorescence images of ZM-positive and ZM-negative sGBM. MET is labeled in yellow, FAM3C in red, PCDHGA3 in green, and nuclei are counterstained with DAPI (blue). Merged images show the spatial co-expression of the three biomarkers. Compared with ZM-negative tumors, ZM-positive tumors displayed stronger fluorescence signals for MET, FAM3C, and PCDHGA3. **b** Representative conventional chromogenic immunohistochemistry images of MET, FAM3C, and PCDHGA3 in ZM-positive and ZM-negative tumors. Low-magnification (10 ×) and high-magnification (40 ×) images show stronger staining of all three markers in ZM-positive tumors compared with ZM-negative tumors. Scale bars are shown in each panel

To corroborate the transcriptomic biomarkers at the protein level, multiplex immunohistochemistry was performed on an independent FFPE validation cohort comprising ZM-positive and ZM-negative tumors. Consistent with the RNA-seq results, ZM-positive tumor sections displayed strong and spatially co-localized expression of MET, PCDHGA3, and FAM3C, whereas ZM-negative tumors showed substantially weaker or minimal staining signals for these markers (Fig. 6a, Fig. S2).

To further validate these findings using conventional chromogenic immunohistochemistry, we performed single-marker staining for MET, FAM3C, and PCDHGA3 in a subset of ZM-positive and ZM-negative cases. For each case, serial sections from the same tumor level were stained separately for the three markers. Representative low- and high-magnification images showed stronger immunohistochemical staining for MET, FAM3C, and PCDHGA3 in the ZM-positive tumor compared with the ZM-negative tumor (Fig. 6b).

Together, these multiplex and conventional immunohistochemical findings provide protein-level support for the transcriptional upregulation of MET, PCDHGA3, and FAM3C in ZM-positive tumors, reinforcing the potential utility of this three-biomarker panel for identifying ZM fusion-positive sGBM.

## Discussion

In this study, we systematically characterized the molecular landscape and clinical significance of the PTPRZ1-MET fusion in sGBM. Six ZM fusion variants were identified, including four novel forms involving PTPRZ1 exons 4, 7, 11, and 12 fused to exon 2 of MET, thereby expanding the known repertoire of this oncogenic rearrangement. ZM fusion occurred more frequently in younger patients and was associated with distinct clinical features, including a lower frequency of seizure presentation. These observations suggest that ZM-positive tumors may represent a biologically and clinically distinct subgroup of sGBM. Importantly, patients harboring ZM fusions exhibited significantly shorter OS and PFS compared with ZM-negative patients, reaffirming the fusion as a strong adverse prognostic factor.

Transcriptomic profiling revealed that ZM-positive tumors are characterized by a proliferation-dominant gene expression program, marked by the upregulation of pathways related to cell-cycle progression, DNA replication stress, homologous recombination repair, and mitotic spindle organization. These features are commonly associated with increased proliferative capacity and genomic instability in high-grade gliomas, providing a mechanistic basis for the aggressive clinical behavior observed in ZM-positive tumors. The enrichment of hallmark pathways such as E2F targets, G2/M checkpoint, and MYC activation further suggests that ZM fusion may drive transcriptional networks that accelerate cell-cycle progression and tumor growth.

Despite the clinical relevance of the ZM fusion, its detection in routine diagnostic practice remains challenging. Although our group first reported this fusion event in sGBM, reliable identification is hindered by several technical limitations. No antibody currently exists that specifically recognizes the ZM fusion breakpoint, and antibodies targeting PTPRZ1 or MET alone lack sufficient specificity, limiting their utility for direct fusion-specific detection. Fluorescence in situ hybridization (FISH) offers an alternative approach but is time-consuming, sensitive to intratumoral spatial heterogeneity, and prone to false-positive or false-negative results due to probe design or tissue quality constraints [18, 19]. RT-PCR followed by Sanger sequencing remains the most analytically precise method but is not readily scalable for routine or high-throughput use [17]. Together, these obstacles highlight the need for surrogate biomarkers capable of reliably identifying ZM-positive tumors in a clinically feasible manner. Compared with existing ZM detection approaches, the MET/PCDHGA3/FAM3C panel has several potential advantages. Sequencing-based or RT-PCR/Sanger-based methods remain definitive for confirming ZM fusion status, but they require high-quality nucleic acids, specialized platforms, and additional turnaround time. In contrast, because MET, PCDHGA3, and FAM3C can be evaluated by immunohistochemistry on FFPE sections, this panel may provide a pathology-compatible screening strategy to identify tumors with a high likelihood of ZM fusion. Moreover, the combination of three markers reduces reliance on MET alone and captures complementary biological features of ZM-positive tumors, including oncogenic signaling, altered adhesion, and microenvironmental remodeling.

To address this unmet diagnostic need, we applied a machine-learning-based feature-prioritization strategy to identify transcriptomic biomarkers associated with ZM fusion status. Rather than relying on a single classifier, we benchmarked eight machine-learning algorithms using cross-validated out-of-fold predictions. XGBoost showed competitive discriminative performance among the evaluated classifiers and provided an interpretable gain-based feature-importance framework. Using this approach, MET, PCDHGA3, and FAM3C were prioritized as candidate biomarkers. Importantly, the combined three-gene panel outperformed individual markers and pairwise combinations in the XGBoost framework, supporting the incremental value of integrating these biomarkers rather than relying on a single marker such as MET alone.

Several additional analyses were performed to assess the robustness of this biomarker selection strategy. First, age- and sex-adjusted differential expression analysis showed that MET, PCDHGA3, and FAM3C remained significantly upregulated in ZM-positive tumors, indicating that their expression differences were not solely attributable to demographic imbalance. Second, sensitivity analyses across alternative candidate gene-set sizes demonstrated that the overall XGBoost performance remained stable across Top20, Top30, Top40, Top50, and Top100 feature pools. MET was consistently retained as a top-ranked feature, whereas PCDHGA3 and FAM3C were repeatedly recovered among high-priority features in Top30 and larger candidate pools. Third, because ZM-positive cases were underrepresented, we further evaluated class-weighted XGBoost. The class-weighted model achieved comparable ROC-AUC and improved sensitivity and balanced accuracy, supporting the stability of the three-gene panel under imbalance-aware modeling. Together, these findings suggest that the MET/PCDHGA3/FAM3C panel was not an artifact of a single feature cutoff, random seed, demographic imbalance, or class-imbalance effect.

Among these markers, MET is a well-established proto-oncogene activated through amplification, mutations, rearrangements, and transcriptional upregulation in diverse malignancies [20–22]. Aberrant MET signaling promotes tumor proliferation, migration, invasion, and resistance to apoptosis [23, 24]. In ZM fusion, MET constitutes the C-terminal kinase domain, and its elevated expression in ZM-positive tumors aligns with persistent pathway activation and fusion-driven oncogenic signaling.

PCDHGA3, a member of the protocadherin gamma subfamily, is known for its roles in neural development and cardiovascular disorders [25]. Although its involvement in gliomas has not been previously characterized, prior studies have reported PCDHGA3 alterations in CNS-PNET and pineoblastoma [26], and dysregulation in ischemic cardiomyopathy resulting in disrupted intercellular junctions and electrical instability [27]. These findings suggest possible roles in maintaining tissue architecture or modulating cellular interactions, warranting further investigation in glioma biology. In ZM-positive tumors, the upregulation of PCDHGA3 may reflect altered adhesion-related or tissue-architecture programs associated with fusion-driven tumor progression, although its functional contribution remains to be experimentally defined.

FAM3C has recently been recognized as an oncogenic driver in multiple solid tumors [28]. It promotes epithelial-mesenchymal transition (EMT), extracellular matrix remodeling, and metastatic dissemination, and its upregulation is linked to poor prognosis [28–30]. Co-amplification and co-expression of FAM3C and MET were shown to synergistically enhance invasive potential through MMP2/9 activation [31]. FAM3C derived from cancer-associated adipocytes further modulates the tumor microenvironment through anti-apoptotic and anti-fibrotic signaling [32], and exosomal transfer of FAM3C activates Src/STAT3 signaling in recipient cells [30]. Although its role in gliomas remains incompletely defined, our findings suggest that FAM3C may participate in ZM-driven oncogenic networks, potentially contributing to EMT-like programs, microenvironmental remodeling, or intercellular communication. Together, the coordinated elevation of MET, PCDHGA3, and FAM3C may capture complementary biological features of ZM-positive tumors, including oncogenic signaling, altered adhesion, and microenvironmental remodeling.

Protein-level validation further supported the transcriptomic findings. Multiplex immunohistochemistry demonstrated stronger co-expression signals of MET, PCDHGA3, and FAM3C in ZM-positive tumors than in ZM-negative tumors, and conventional chromogenic immunohistochemistry provided additional visual support for stronger staining of these markers in representative ZM-positive samples. Importantly, the machine-learning classifier was developed using RNA-seq-derived mRNA expression, whereas the immunohistochemical analyses were intended as orthogonal protein-level validation rather than an interchangeable diagnostic assay. These findings reinforce the biological relevance and potential pathology-compatible applicability of the transcriptomic biomarker panel. Nevertheless, because the current immunohistochemical analyses were primarily qualitative, future studies incorporating standardized staining protocols, quantitative scoring systems, predefined positivity thresholds, digital image quantification, and larger independent validation cohorts will be needed before clinical implementation.

Several limitations should be acknowledged. First, this was a retrospective study with a relatively small number of ZM-positive cases, reflecting the low prevalence of this fusion event. Although stratified cross-validation, repeated random-seed analyses, feature-pool sensitivity analyses, and class-weighted modeling were used to improve robustness, external validation in larger multicenter cohorts remains necessary. Second, because ZM fusion is mainly relevant to tumors historically referred to as secondary glioblastomas, the MET/PCDHGA3/FAM3C panel should be interpreted within this specific disease context rather than as a generalizable classifier for other glioma subtypes or other cancer types. Third, the top-ranked biomarkers were identified through association and classification analyses, and their functional roles in ZM-driven gliomagenesis require further experimental validation.

In summary, the three-gene panel composed of MET, PCDHGA3, and FAM3C represents a promising molecular tool for distinguishing ZM-positive from ZM-negative sGBM and provides new insight into the biology of ZM fusion-driven gliomagenesis. These findings provide a basis for further validation of biomarker-guided diagnostic strategies and for mechanistic studies of ZM fusion-driven tumor progression in sGBM.

## Materials and methods

### Patient cohort and ethics approval

Fresh-frozen tumor tissues from 159 patients classified as clinically defined sGBM according to the original CGGA clinical and pathological annotations were collected at Beijing Tiantan Hospital, Capital Medical University (Beijing, China). Among these cases, 15 tumors harbored the PTPRZ1-MET fusion, while 144 were classified as ZM-negative. Patients were included if they were classified as sGBM according to the original CGGA clinical and molecular annotations, had available fresh-frozen tumor tissue for RNA sequencing, available ZM fusion status, and corresponding clinicopathological information (Table S7). Cases were excluded if tumor tissue quality was insufficient for RNA sequencing, key molecular or clinical annotations were unavailable, or the available longitudinal clinical and pathological records did not support a clinically defined sGBM disease course. Tumor specimens were obtained at the time of surgical resection, immediately snap-frozen in liquid nitrogen to preserve RNA integrity until further processing. Comprehensive clinical information–including diagnosis, age, sex, WHO grade, progression-free survival (PFS), overall survival (OS)–was systematically curated along with matched tissue samples and integrated into the Chinese Glioma Genome Atlas (CGGA) platform, as described previously [33, 34]. Clinical follow-up data in the CGGA cohort were continuously maintained and updated at 6-month intervals. Overall survival was defined as the time from surgery to death or last follow-up, and progression-free survival was defined as the time from surgery to tumor progression, death, or last follow-up. Patients without an event at the last follow-up were censored. All procedures were conducted in accordance with the ethical standards of the Declaration of Helsinki and were approved by the Institutional Review Board of Beijing Tiantan Hospital. Written informed consent was obtained from all patients prior to enrollment.

### RNA sequencing and sanger sequencing

Total RNA was extracted from fresh-frozen tumor tissues using TRIzol reagent (Invitrogen) and quantified using a NanoDrop spectrophotometer. RNA integrity was assessed with an Agilent 2100 Bioanalyzer, and samples with RNA integrity number (RIN) ≥ 7.0 were used for sequencing. mRNA libraries were prepared using the TruSeq Stranded mRNA Library Prep Kit (Illumina), followed by poly(A) selection and fragmentation. Paired-end 101-bp sequencing was performed on the Illumina HiSeq 4000 platform.

RNA-seq data were used for fusion transcript detection, and candidate PTPRZ1-MET fusions were identified using *STAR-Fusion*. Fusion calls were manually reviewed based on supporting junction reads, spanning reads, and annotated breakpoint information. For validation of representative PTPRZ1-MET fusion events, reverse transcription PCR (RT-PCR) followed by Sanger sequencing was performed. Primers flanking the ZM fusion breakpoint (forward: 5'-CCGTCTGGAAATGCGAATCCTAAA-3'; reverse: 5'-CAGGCCCAGTCTTGTACTCAGCAA-3') were designed to amplify reported ZM fusion variants. PCR amplification was carried out using GoTaq DNA polymerase (Promega), and products were resolved by agarose gel electrophoresis, purified, and subjected to Sanger sequencing. The resulting chromatograms were manually inspected and aligned to reference PTPRZ1-MET junction sequences to confirm the presence and specific subtype of the fusion when applicable. Representative Sanger sequencing traces are provided in Fig. S3.

### Clinical characteristics, fusion-variant distribution, and survival analysis

Baseline clinicopathological characteristics, including age at diagnosis, sex, extent of resection, tumor location, laterality, and seizure status, were compared between ZM-positive and ZM-negative patients. Continuous variables were summarized as mean ± standard deviation or median with range, as appropriate, and categorical variables were summarized as number and percentage. Tumor locations were recorded according to involved anatomical regions and were not treated as mutually exclusive when more than one lobe was involved. Extent of resection and laterality were treated as mutually exclusive categories, and missing values were reported explicitly. For age-stratified analyses, patients were grouped using 40 years as the cutoff. The distribution of ZM fusion variants was summarized according to the PTPRZ1 exon involved in the fusion junction, with all detected variants sharing MET exon 2 as the downstream fusion partner.

OS and PFS were analyzed according to the definitions described above. Kaplan–Meier survival curves were generated to compare survival outcomes between ZM-positive and ZM-negative groups, and statistical significance was assessed using the log-rank test with the R package *survival* (v3.7–0).

### Differential gene expression and heatmap visualization

Raw sequencing reads were aligned to the human reference genome (GRCh38) using *STAR* (v2.7.9a), and gene-level expression matrices were generated for downstream analyses. Normalized RNA-seq expression values were log2-transformed before statistical testing. ZM-associated candidate genes were identified using gene-wise Welch’s two-sample t tests comparing ZM-positive and ZM-negative tumors, with fold change calculated as the ratio of mean normalized expression between the two groups. Genes with fold change > 2 and nominal *P* < 0.05 were retained for downstream functional analysis and feature discovery. To assess potential confounding by age or sex, unadjusted and age/sex-adjusted *limma* empirical Bayes linear models were further fitted to the log2-transformed expression matrix, with ZM fusion status specified as the primary variable of interest. For machine-learning feature discovery, nominally significant ZM-associated genes were ranked by fold-change magnitude, and a balanced 40-gene candidate feature pool was constructed by selecting the top 20 upregulated and top 20 downregulated genes in ZM-positive tumors. The top 20 upregulated genes were visualized using the *pheatmap* package (v1.0.12) with row-wise z-score scaling.

### Functional enrichment analysis

A total of 359 upregulated differentially expressed genes in the ZM-positive group were subjected to GO and KEGG enrichment analyses using the DAVID bioinformatics resource (https://davidbioinformatics.nih.gov/). Enrichment terms with Benjamini-Hochberg–adjusted *p* < 0.05 were considered statistically significant. GO enrichment results were summarized across the biological process (BP), molecular function (MF), and cellular component (CC) categories. Enrichment pathways and functional terms were visualized in R using the *ggplot2* package (v3.5.1) with bar-plot and dot-plot representations.

### Gene set enrichment analysis

GSEA was conducted using the GSEA Java application (v4.1.0) with the Hallmark curated gene sets from the Molecular Signatures Database (MSigDB v7.4). Genes were ranked by log₂ fold change between ZM-positive and ZM-negative tumors, and enrichment significance was evaluated using 1,000 phenotype permutations. Pathways with a false discovery rate (FDR) < 0.25 were considered significantly enriched. Leading-edge analysis was performed to identify the core gene sets driving the enrichment signal for each significant pathway.

### Machine learning for biomarker selection

Machine-learning analyses were performed to prioritize transcriptomic biomarkers capable of distinguishing ZM-positive from ZM-negative tumors. The predefined Top40 candidate gene set described above was used as the initial input. Eight classification algorithms were evaluated, including XGBoost, random forest, LASSO-regularized logistic regression, support vector machine, logistic regression, gradient boosting machine, AdaBoost, and naive Bayes. For each algorithm, model-specific feature-ranking, coefficient-based ranking, or variable-importance measures were used to identify the top three model-prioritized features from the Top40 candidate gene set. These model-specific top-ranked features were then used to construct the corresponding three-gene classifier.

All models were trained using the caret framework with stratified fivefold cross-validation. Hyperparameters were optimized using predefined grid searches, and cross-validated out-of-fold predicted probabilities were used to generate receiver operating characteristic (ROC) curves and calculate area under the curve (AUC) values. XGBoost was retained as the primary feature-prioritization model because it showed competitive discriminative performance among the evaluated algorithms and provided gain-based feature importance while capturing nonlinear relationships and feature interactions. For the XGBoost model, the hyperparameter grid included *nrounds* = 100 or 200, *max_depth* = 3 or 6, eta = 0.1, *gamma* = 0 or 0.1, *colsample_bytree* = 0.8, *min_child_weight* = 1 or 3, and *subsample* = 0.8. The top-ranked XGBoost features were further evaluated using single-gene, pairwise, and combined three-gene models. Detailed hyperparameter grids and performance metrics for the eight machine-learning classifiers are provided in Table S8.

To further minimize potential information leakage during feature selection and model evaluation, a fully nested stratified cross-validation analysis was performed. In each outer training fold, differential expression filtering, Top40 candidate gene selection based on the top 20 upregulated and top 20 downregulated genes, XGBoost feature prioritization, hyperparameter tuning, and model fitting were repeated using only the outer training samples. The held-out outer fold was not used for differential expression analysis, feature selection, hyperparameter optimization, or threshold determination, and was used exclusively for performance evaluation. Outer-fold predicted probabilities were pooled to calculate ROC-AUC, sensitivity, specificity, positive predictive value, negative predictive value, accuracy, balanced accuracy, and their 95% confidence intervals. In this nested framework, both the training-fold Top40 model and the training-fold-selected Top3 model were evaluated. In addition, the final fixed MET/PCDHGA3/FAM3C panel was evaluated within the same outer-loop framework by training the model on each outer training fold and predicting the corresponding held-out fold without reselecting the biomarkers.

To evaluate the robustness of the feature-pool size and feature-prioritization strategy, sensitivity analyses were performed using alternative candidate gene-set sizes, including Top20, Top30, Top40, Top50, and Top100 genes, generated using the same ranking strategy. For each candidate set, XGBoost models were trained using stratified fivefold cross-validation repeated across 50 random seeds, and model performance was summarized as mean AUC ± standard deviation. The inclusion status and feature-importance ranks of MET, PCDHGA3, and FAM3C were recorded across candidate gene-set sizes. To account for the unequal number of ZM-positive and ZM-negative cases, class-weighted XGBoost was further evaluated as a sensitivity analysis using the fixed MET/PCDHGA3/FAM3C panel. In the weighted model, ZM-positive samples were assigned a weight equal to the ratio of ZM-negative to ZM-positive cases, whereas ZM-negative samples were assigned a weight of 1. Decision curve analysis was performed using cross-validated out-of-fold predicted probabilities from the XGBoost model to evaluate the potential clinical net benefit compared with the “all” and “none” strategies.

### Multiplex immunohistochemistry and conventional immunohistochemistry on paraffin-embedded sections

An independent FFPE validation cohort consisting of ZM-positive (n = 14) and ZM-negative (n = 10) tumors was obtained from the Department of Pathology, Beijing Tiantan Hospital, Capital Medical University. ZM fusion status and integrated pathological diagnosis in this validation cohort were determined by postoperative molecular pathological testing before inclusion in this study. The clinicopathological information of this independent validation cohort is summarized in Table S9. For multiplex immunohistochemistry, FFPE samples from 10 ZM-positive and 6 ZM-negative tumors were stained using a fluorescence-based detection kit (ImmunoWay, Cat. No. RS0035) according to the manufacturer’s instructions. Primary antibodies included MET (Cell Signaling Technology, Cat. No. 8198S), PCDHGA3 (Thermo Fisher Scientific, Cat. No. PA5-71,834), and FAM3C (Proteintech, Cat. No. 60282–1-Ig). Fluorescent signals were visualized using a fluorescence imaging system, and biomarker expression intensity and spatial localization were qualitatively assessed.

To further validate the expression patterns of the three candidate biomarkers by conventional immunohistochemistry, additional FFPE samples from 4 ZM-positive and 4 ZM-negative tumors within the same independent validation cohort were selected for single-marker staining. For each patient, three serial sections from the same tumor level were prepared and separately stained for MET, PCDHGA3, and FAM3C using the same primary antibodies described above. Immunohistochemical staining was performed using standard chromogenic procedures, and the expression patterns of each marker were compared between ZM-positive and ZM-negative tumors.

### Statistical analysis

All statistical analyses were conducted in R (v4.4.3). Continuous variables were compared using Student’s t-test or the Mann–Whitney U test, as appropriate, and categorical variables were compared using the χ^2^ test or Fisher’s exact test. Kaplan–Meier survival analysis with the log-rank test was used to compare overall survival and progression-free survival between groups. Differential expression analyses incorporated Benjamini–Hochberg correction to control the false discovery rate. For machine-learning analyses, receiver operating characteristic (ROC) curves and area under the curve (AUC) values were calculated using cross-validated out-of-fold predicted probabilities. Representative ROC curves were generated from one stratified fivefold cross-validation run using a fixed random seed. For robustness analyses, cross-validation was repeated across 50 random seeds, and model performance was summarized as mean ± standard deviation. For class-weighted XGBoost analyses, PR-AUC, sensitivity, specificity, F1 score, and balanced accuracy were also reported. Unless otherwise specified, all statistical tests were two-sided, and *p* < 0.05 was considered statistically significant.

## Supplementary Information

Supplementary Material 1.

Supplementary Material 2.

## Data Availability

The raw RNA-seq data have been deposited in the Genome Sequence Archive for Human (GSA-Human) at CNCB-NGDC under accession number HRA019727. The dataset is available under controlled access through GSA-Human. Representative Sanger sequencing data supporting the validation of selected PTPRZ1-MET fusion junctions are provided in the Supplementary Information. Additional supporting data and materials are available from the corresponding author upon reasonable request.
