# Supplementary material for: Artificial intelligence-based diagnostic model for identifying PTPRZ1-MET fusion in a clinically defined secondary glioblastoma cohort
Source: Mol Biomed. 2026 Aug 4;7:124. doi: 10.1186/s43556-026-00529-x (PMC13433701; doi:10.1186/s43556-026-00529-x)
Supplement: Supplementary file 1 [file 43556_2026_529_MOESM1_ESM.docx]

**Artificial intelligence-based diagnostic model for identifying PTPRZ1-MET fusion in a clinically defined secondary glioblastoma cohort**

Jin-Hao Zhang^1,†^, Ji Shi^2,3,†^, Han-Xiao Zhou^1^, Wen-Lu Tan^1^, Ying Zhang^1,*^, Tao Jiang^1,2,4,5,*^, Zheng Zhao^1,4,5,*^

^1^Department of AI and Biomedical Information, Beijing Neurosurgical Institute, Capital Medical University, Beijing 100070, China

^2^Department of Neurosurgery, Beijing Tiantan Hospital, Capital Medical University, Beijing 100070, China

^3^ Department of Neurosurgery, Liaoning Cancer Hospital & Institute, Cancer Hospital of Dalian University of Technology, Cancer Hospital of China Medical University, Shenyang 110042, China

^4^Chinese/Asian Glioma Genome Atlas (CGGA/AGGA) Network, Beijing 100070, China

^5^Chinese Neuro-Oncology Genome Atlas (CGGA-CNS) Network, Beijing 100070, China

^*^Corresponding author: [zhaozheng0503@ccmu.edu.cn](mailto:zhaozheng0503@ccmu.edu.cn) (Zheng Zhao), [taojiang1964@163.com](mailto:taojiang1964@163.com) (Tao Jiang) and [zhangy_bni@163.com](mailto:zhangy_bni@163.com) (Ying Zhang)

^†^These authors contributed equally to this work

SUPPLEMENTARY FIGURE

**Fig. S1** **Additional performance and robustness analyses of machine-learning models for ZM fusion classification. (a)** Confusion matrices of eight model-specific three-gene classifiers, including random forest, logistic regression, support vector machine, naive Bayes, LASSO, AdaBoost, gradient boosting machine, and XGBoost. Predicted and true labels are shown for ZM-positive and ZM-negative tumors. **(b)** Decision curve analysis of the XGBoost model based on cross-validated out-of-fold predicted probabilities, compared with the “all” and “none” strategies. **(c)** Outer-loop ROC curves from fully nested cross-validation, showing the performance of the training-fold Top40 model, training-fold-selected Top3 model, and fixed MET/PCDHGA3/FAM3C panel. **(d)** XGBoost performance across different candidate feature-pool sizes, including Top20, Top30, Top40, Top50, and Top100 genes. Points indicate mean cross-validated AUC values across repeated random seeds, and error bars indicate standard deviations.

**Fig. S2** **Additional multiplex immunofluorescence validation of MET, FAM3C, and PCDHGA3 expression in independent FFPE tumor samples.** Representative merged multiplex immunofluorescence images are shown for additional ZM-positive cases (cases 5–12) and ZM-negative cases (cases 13–16). DAPI marks nuclei, whereas MET, FAM3C, and PCDHGA3 are shown in yellow, red, and green, respectively. ZM-positive tumors showed stronger combined staining signals for the three biomarkers than ZM-negative tumors. Scale bars = 50 μm.

**Fig. S3** **Representative Sanger sequencing chromatograms validating selected PTPRZ1-MET fusion junctions.** Sanger sequencing traces showing PTPRZ1-MET fusion breakpoints in three representative fusion-positive cases: PTPRZ1 exon 4–MET exon 2 in CGGA_sGBM_P45 **(a)**, PTPRZ1 exon 3–MET exon 2 in CGGA_sGBM_P54 **(b)**, and PTPRZ1 exon 1–MET exon 2 in CGGA_sGBM_P85 **(c)**. The red vertical line indicates the fusion junction. Orange and blue bars indicate PTPRZ1-derived and MET-derived sequences, respectively.

**SUPPLEMENTARY TABLES**

Supplementary Tables S1–S9 are provided in the accompanying Excel file.

Table S1. Age- and sex-adjusted limma analysis of the original ZM-upregulated transcriptional program.

Table S2. Cross-validated performance metrics of eight machine-learning classifiers based on model-specific three-gene panels derived from the Top40 candidate gene set.

Table S3. Age- and sex-adjusted differential expression analysis of MET, PCDHGA3, and FAM3C between ZM-positive and ZM-negative tumors.

Table S4. Performance metrics of fully nested cross-validation analyses.

Table S5. XGBoost feature-pool sensitivity analysis across Top20, Top30, Top40, Top50, and Top100 candidate gene sets.

Table S6. Performance comparison of unweighted and class-weighted XGBoost models using the fixed MET/PCDHGA3/FAM3C panel.

Table S7. Case-level clinicopathological characteristics and prior lower-grade glioma history of the 159 secondary glioblastoma cases.

Table S8. Hyperparameter grids of the eight machine-learning classifiers evaluated in this study.

Table S9. Clinicopathological characteristics and validation assays of the independent FFPE cohort used for protein-level validation.
